# Molecular heterogeneity of HPV-associated cancers and strategies to overcome treatment resistance

**DOI:** 10.47248/chp2603010002

**Published:** 2026-03-01

**Authors:** Sara Rasouli, Weiyi Gong, Raegan Wood, Danyal Daneshdoust, Anam Khan, Rani Mahyoob, Chongwen Cao, Zihao Yu, Nagireddy Putluri, Gennady Shvets, Haichang Li, Bo Zhao, Xuefeng Liu, Jenny Li

**Affiliations:** 1. Comprehensive Cancer Center, The Ohio State University, Columbus, OH, 43210, USA; 2. Biomedical Science Graduate Program (BSGP), College of Medicine, The Ohio State University, Columbus, OH, 43210, USA; 3. Medical Scientist Training Program (MSTP), College of Medicine, The Ohio State University, Columbus, OH, 43210, USA; 4. Department of Molecular and Cellular Biology, Baylor College of Medicine, Houston, TX, 77030 USA; 5. School of Applied and Engineering Physics, Cornell University, Ithaca, NY, 14853, USA; 6. Department of Veterinary Biosciences, The Ohio State University College of Veterinary Medicine, Columbus, OH, 43210, USA; 7. Division of Infectious Disease, Department of Medicine, Brigham and Women’s Hospital, Harvard Medical School, Boston, MA, 02115, USA; 8. Departments of Pathology, Urology and Radiation Oncology, Wexner Medical Center, The Ohio State University, Columbus, OH, 43210, USA; 9. Department of Pathology, Wexner Medical Center, The Ohio State University, Columbus, OH, 43210, USA

**Keywords:** HP, viral genome integration, heterogeneity, treatment

## Abstract

Human papillomavirus (HPV) is a major driver of global cancer incidence, responsible for nearly all cervical cancers and a significant proportion of oropharyngeal, anal, and other anogenital cancers. Despite the availability of effective vaccines, HPV-associated cancers persist due to persistent infection, immune evasion, and the virus’s ability to integrate into the host genome, contributing to molecular heterogeneity and therapeutic resistance. Here, we summarize molecular heterogeneity and the emerging combined therapeutic strategies. High-risk HPV types, notably HPV16 and HPV18, initiate carcinogenesis through persistent infection of epithelial basal cells and through the actions of the E6 and E7 oncoproteins, which disrupt the p53 and Rb pathways, induce telomerase activation, and promote genomic instability. A critical step in the progression to cancer is HPV genome integration, which occurs through DNA damage response pathways and results in heterogeneous insertion patterns that drive oncogene activation, tumor suppressor inactivation, and complex chromosomal rearrangements. Emerging single-cell RNA sequencing studies have highlighted the transcriptional, immune, and spatial heterogeneity of HPV-associated cancers, revealing subpopulations linked to immune escape, therapy resistance, and disease progression. These technologies have uncovered dynamic microenvironmental shifts and distinct immune cell populations, underscoring the importance of cellular and spatial heterogeneity in shaping tumor evolution and treatment response. The “hit and run” hypothesis suggests that while HPV oncoproteins are critical in early carcinogenesis, some tumors may lose dependence on viral oncogenes as they accumulate host genomic alterations, complicating detection and treatment strategies. Heterogeneity in HPV integration patterns and the tumor microenvironment contribute to variable treatment outcomes and the development of resistance to monotherapies. To overcome the challenges posed by molecular heterogeneity in HPV-associated cancers, combined therapeutic strategies targeting viral oncoproteins, host genomic vulnerabilities, and immune microenvironment are essential. Integrating single-cell transcriptomic insights with HPV integration profiles highlights how viral and host heterogeneity shape immune escape and therapeutic response, offering a framework for designing personalized combination therapies to improve outcomes in HPV-associated cancers.

## 1. Introduction

Human papillomavirus (HPV) is one of the most widespread cancer-causing viruses, playing a significant role in the global incidence of malignancies. Persistent HPV infection is estimated to contribute to about 5% of all cancer cases worldwide, leading to over 630,000 new diagnoses each year. In the United States, HPV-associated cancers account for approximately 45,000 new cases annually, with close to 60% diagnosed in women and 40% in men. HPV is identified as the primary cause of nearly all cervical cancers (99%), the majority of anal cancers (90%), and a significant proportion of oropharyngeal (70%), vaginal, vulvar, and penile cancers [1,2].

Although effective vaccines are available to prevent HPV infection, HPV-driven cancers continue to be a serious public health issue. This ongoing challenge is largely driven by the genetic heterogeneity of HPV, individual differences in immune mechanisms, and the virus’s various strategies for integrating into host cells [3]. In contrast to most viruses, HPV-driven oncogenesis occurs gradually through cellular changes that can take years or even decades. HPV establishes a latent infection by infecting basal epithelial cells, mostly through mucosal microabrasions. Through complex pathways, HPV gradually eludes immune control, enabling cellular dysregulation, genomic integration, and persistent infection, all of which eventually contribute to the development of cancer. The most effective way for this virus to be transmitted is through epithelial contact. Although the immune system eliminates most HPV infections, a part of it remains, especially in those who have immunosuppressive conditions [4]. The major challenge in HPV-associated cancers is their significant heterogeneity. Additionally, HPV integration into the host genome is not uniform across cancer types, affecting gene expression, chromosomal instability, and tumor development and progression [5]. In this review, we explore the diverse molecular processes driving HPV-related tumor development, highlight the cellular heterogeneity revealed by single-cell approaches, and discuss innovative combinatorial treatments aimed at addressing resistance and improving therapeutic efficacy.

## 2. Differences Between Cervical and Head-and-Neck HPV Carcinogenesis

While the fundamental oncogenic mechanism-expression of the viral oncoproteins E6 and E7is shared between Human Papillomavirus (HPV)-driven cervical cancer (CC) and HPV-positive Head-and-Neck Squamous Cell Carcinoma (HNSCC), key differences in viral integration patterns, co-mutational landscapes, and host-virus interactions dictate distinct biological behaviors and clinical outcomes. These differences trace back to the distinct roles HPV plays in the onset and progression of the two cancer types.

The epidemiological and etiologic paths diverge significantly. Cervical cancer (CC) development is typically characterized by a well-defined progression from pre-invasive lesions (cervical intraepithelial neoplasia, or CIN) over many years [6], frequently requiring co-factors such as chronic inflammation or coinfection, where HPV is necessary but often not the sole initiator [6]. In sharp contrast, HPV-positive Oropharyngeal Squamous Cell Carcinoma (OPSCC) often arises de novo in the tonsils or base of the tongue, and HPV infection is considered the primary and largely sufficient oncogenic event, typically overriding the traditional risk factors of tobacco and alcohol use associated with HPV-negative HNSCC [7].

This difference in the virus’s role is also reflected at the genomic level. In CC, stable, high-level expression of E6 and E7 is achieved and maintained through the complete integration of the HPV genome into the host cell DNA, a near-universal event in CC progression [6]. Conversely, HPV-positive OPSCC frequently maintains the HPV genome in a stable episomal (non-integrated) form [7], although integration can also occur. The persistence of episomes or integrated viral DNA in HNSCC drives carcinogenesis, but the resulting mutational signature differs significantly from that of CC, often featuring markedly fewer total genomic alterations [7].

This difference in viral-host interaction extends to specific signaling pathways and therapeutic vulnerabilities. In cervical cancer (CC), HPV-positive cells rely heavily on pathways such as the PI3K/AKT/mTOR signaling cascade [6], which has been shown to be crucial for regulating viral-host crosstalk, survival, and proliferation. In contrast, HPV-positive OPSCCs demonstrate different dependencies and adaptations. Studies focusing on the Head-and-Neck site have specifically indicated that the presence of HPV leads to the activation of specific DNA damage repair (DDR) factors in oropharyngeal cancers [8]. This activation suggests that these tumors possess an altered response to DNA damage [9] that may influence their sensitivity to chemo- and radiotherapy compared to HPV-negative tumors [10]. Furthermore, the host immune microenvironment is markedly different; HPV-positive HNSCCs are generally considered immunologically “hotter” tumors, characterized by a higher density of tumor-infiltrating lymphocytes [7], which is strongly associated with a favorable prognosis and superior response to immune checkpoint blockade compared to both CC and HPV-negative HNSCC. These molecular, etiological, and immunological variances necessitate site-specific therapeutic strategies despite the shared viral etiology.

## 3. Challenges in Screening and Persistent Infection

The clinical significance of HPV infection status and the challenges associated with screening are profoundly different between the cervical and head-andneck sites, directly impacting patient management and preventative strategy.

Cervical Cancer (CC) Screening: Screening for CC is well established and effective, relying on routine cytology (Pap smears) and HPV DNA testing [6,11]. This established system is effective because CC progresses slowly through well-defined, detectable precursor lesions (CIN) [6]. The primary goal of cervical screening is the early detection and treatment of these precursors to prevent invasive cancer. A key challenge remains the management of transient *versus* persistent infection; while most HPV infections are transient and cleared by the immune system, the persistence of high-risk HPV infection is the necessary precursor for progression to CIN and, eventually, cervical cancer [6]. The difficulty lies in accurately identifying which persistent infections will progress to malignancy, as molecular markers for this transition remain an area of intense study [12].

Head-and-Neck Squamous Cell Carcinoma (HNSCC) Challenges: In contrast, there is currently no established or clinically viable screening program for HPV-positive Oropharyngeal Squamous Cell Carcinoma (OPSCC) [10]. Unlike cervical cancer, OPSCC often appears to arise *de novo*, skipping a long, detectable pre-invasive phase, or with precursor lesions (such as altered tonsillar crypts) that are difficult to visualize and sample [10]. Furthermore, HPV is ubiquitous, meaning the detection of HPV DNA or even high-risk HPV types in the oral cavity or oropharynx does not reliably predict cancer development, as transient infection is extremely common in the general population [10]. The challenge lies in identifying the small fraction of persistent, high-risk infections that will progress to malignancy versus the large number of clinically irrelevant, transient infections [12]. Additionally, while the long-term persistence of HPV-positive HNSCC is highly treatable, primary prevention relies solely on HPV vaccination, emphasizing the importance of broader immunization efforts.

Prognosis as a Clinical Challenge: Perhaps the most striking difference between the two cancer types is their clinical prognosis. Cervical cancer (CC), historically, has a poorer overall prognosis compared to HPV-negative HNSCC, especially in advanced stages, requiring aggressive multimodal therapy [6]. This outcome is often attributed to the high genomic instability and complex mutational landscape characteristics of CC [6]. Conversely, the presence of HPV in Oropharyngeal Squamous Cell Carcinoma (OPSCC) is the single strongest predictor of a favorable clinical outcome [10]. HPV-positive OPSCC patients demonstrate significantly higher response rates to chemoradiation and superior overall survival compared to their HPV-negative counterparts [10].

Treatment Response and De-escalation: The disparate prognostic profiles drive distinct therapeutic goals. The challenge in CC is to identify novel, targeted therapies, often requiring aggressive multimodal treatment (surgery, radiotherapy, and chemotherapy) to overcome inherent resistance and genomic complexity [6]. For OPSCC, the primary therapeutic challenge is de-escalation: maintaining high cure rates while reducing the intensity of chemoradiation to mitigate debilitating long-term side effects (*e.g*., dysphagia and xerostomia), a strategy enabled by the tumor’s favorable biology and increased radiosensitivity [10].

Vaccine and Primary Prevention: The role of the HPV vaccine also differs in its timeline of clinical impact. Cervical cancer prevention has seen immense success due to decades of organized screening coupled with population-wide HPV vaccination programs [11]. While HNSCC prevention fundamentally relies on the same primary vaccination strategy, the effect is delayed by the longer latency period for OPSCC development and the need for near-universal vaccine uptake to achieve herd immunity across the relevant age groups [13]. The challenge is to ensure high global vaccine coverage to eliminate CC while waiting for the downstream impact on OPSCC incidence.

Monitoring for Recurrence: Post-treatment monitoring presents a unique challenge, particularly regarding the sensitivity and specificity of biomarkers. For CC, monitoring relies heavily on standard imaging and clinical surveillance, though research into molecular recurrence markers is ongoing [6,12]. In contrast, OPSCC monitoring is undergoing a revolutionary shift with the use of circulating tumor HPV DNA (ctDNA). Because OPSCC often sheds high levels of unique viral DNA into the bloodstream, ctDNA provides an exquisitely sensitive, non-invasive method for detecting minimal residual disease and predicting recurrence months before conventional imaging, presenting a significant opportunity for early intervention [10]. The challenge is standardizing this high-sensitivity biomarker for routine clinical use.

## 4. Molecular Mechanisms of HPV Cancer Initiation and Progression

HPV infection is a well-established driver of cancer initiation, particularly in cervical and other anogenital and oropharyngeal cancers. Among the more than 170 HPV types identified to date, only a subset-about 12, including HPV16, HPV18, HPV31, and HPV33-are classified as high-risk types due to their oncogenic potential [13,14]. At the same time, the majority are considered low-risk and rarely cause precancerous changes [15,16]. High-risk HPV types are implicated in over 70% of anal, cervical, vaginal, and vulvar cancers [17].

For high-risk HPV to initiate carcinogenesis, persistent infection is required. Although most high-risk HPV infections are cleared by the immune system within 12–18 months, approximately 10% persist, increasing the risk of progression to cancer [18,19]. HPV infection begins when microabrasions in the stratified squamous epithelium expose the basement membrane, allowing the virus to bind, undergo capsid conformational changes, and infect basal keratinocytes [20]. Once inside, HPV genomes are maintained as episomes, replicating with host DNA and evenly segregating to daughter cells [21,22]. As infected keratinocytes differentiate and migrate toward the epithelial surface, viral genome replication and protein synthesis increase, leading to the assembly and release of new viral particles at the surface.

Because HPV lacks its own DNA polymerase, it depends on the host cell’s S-phase machinery for replication. Differentiated keratinocytes are typically non-proliferative; to facilitate viral replication, HPV expresses the E6 and E7 oncoproteins, which drive these cells into a hyperproliferative state [23]. These oncoproteins are central to HPV-mediated carcinogenesis, functioning synergistically to bypass cell cycle checkpoints [24,25]. E7 binds and inactivates the retinoblastoma protein (pRb), releasing E2F transcription factors and promoting uncontrolled cell cycle progression [24,26,27]. However, this disruption stabilizes p53, triggering apoptosis, which would prevent immortalization [28,29]. E6 counteracts this by binding to and promoting the degradation of p53, thereby inhibiting apoptosis and enabling continued cell proliferation [30,31]. E6 also activates telomerase, helping cells overcome replicative senescence associated with telomere shortening [32,33].

Despite these disruptions, E6 and E7 alone are not sufficient to fully transform epithelial cells. Experimental models indicate that co-expression of E6 and E7 with additional oncogenic factors, such as activated K-Ras or telomerase holoenzyme components, is necessary for malignant transformation [34,35]. *In vivo*, chronic estrogen exposure in conjunction with E6 and E7 expression further facilitates cervical and vaginal squamous cancer initiation [36]. Emerging evidence suggests that heterogeneity in E6 and E7 expression levels may influence persistent infection and contribute to oncogenesis, warranting further study [37].

HPV also reshapes the local microenvironment to support carcinogenesis. E6 and E7 inhibit NF-κB signaling by interacting with its coactivators, thereby suppressing cytokine secretion and dampening type I interferon responses, ultimately promoting immune evasion through increased regulatory T-cell infiltration [38,39]. Additionally, the HPV E5 protein can downregulate MHC class I molecules, impairing antigen presentation and enabling escape from natural killer cell surveillance [40,41].

Progression from low-grade to high-grade squamous intraepithelial lesions and eventually to invasive cancer is closely linked to genomic instability [42], a hallmark of HPV-associated cancers. HPV oncoproteins induce genomic instability by dysregulating the cell cycle, causing DNA damage, generating oxidative stress, and altering telomere dynamics [43,44]. E6-mediated degradation of p53 impairs DNA repair and cell cycle arrest, leading to the accumulation of DNA damage, while E7-mediated inactivation of pRb disrupts cell cycle control, promoting unchecked proliferation. E7 also interacts with cyclins and CDKs, facilitating the transition from the G1 to S phase, and can induce polyploidy and chromosomal abnormalities by disrupting the G2 spindle assembly checkpoint [42,44].

Oxidative stress further contributes to HPV-mediated carcinogenesis [43,45]. Cellular stress conditions such as infection and inflammation disrupt redox homeostasis, leading to the accumulation of reactive oxygen species (ROS), which damage DNA, proteins, and lipids. HPV oncoproteins, including E1, E2, E4, E6, and E7, can disrupt mitochondrial function and promote ROS generation, exacerbating genomic instability [45]. Notably, E6 has been shown to increase ROS levels even in HPV-negative cervical cells [43].

HPV also influences telomere maintenance, an essential aspect of cellular immortality [43,46]. Telomerase, particularly its catalytic subunit TERT, is typically inactive in keratinocytes but can be upregulated by HPV E6, which facilitates the replacement of repressor complexes at the TERT promoter with activators like c-MYC, leading to telomere elongation [43]. E7 can contribute to both telomere shortening and elongation, and in combination with E6, can induce telomere dysfunction, resulting in chromosomal instability and abnormal cell division [43]. E7 can also activate the alternative lengthening of telomeres (ALT) pathway via FANCD2, providing an additional mechanism for telomere maintenance in HPV-associated cancers [43].

E6 and E7 proteins are both essential for host cell immortalization; however, they are not sufficient for full cell transformation and tumorigenesis. This indicates that additional host cell factors are required for complete malignant transformation and progression. Several studies from us and others suggested that constitutive active beta-catenin, Myc, and SV40 small T antigen are sufficient to induce *in vitro* malignant transformation - anchorage independent growth of HPV immortalized keratinocytes [47,48]. In general, no commonly occurring mutations have been identified in cervical cancer initiation or progression [49], suggesting that alternative mechanisms drive malignant transformation. We and others have shown increased telomerase activity in cells immortalized by high-risk HPV E6 and E7 oncogenes through three mechanisms: activation of hTERT, stabilization of hTERT mRNA, and direct interaction with hTERT [50,51]. Consistent with this, TERT expression increases across the cervical dysplasia cascade [52]. Importantly, our previous studies revealed a non-canonical role for TERT in HPV-induced immortalization, whereby TERT regulates cellular gene expression and HPV promoter activity independently of telomerase activity [53,54]. Together with accumulating evidence that both canonical and non-canonical functions of TERT and TERC (the RNA component of the telomerase complex) contribute to cell proliferation, differentiation, and survival, these findings point to telomerase components as active drivers of disease progression. Indeed, TERC is amplified and overexpressed in over 90% of human cervical cancers [55,56]. Moreover, our recent study demonstrated that elevated TERT and TERC expression promote cell growth, anchorage-independent growth, and tumor formation in immunodeficient mice [57], representing a critical step in the conversion of normal to malignant cells.

**Figure 1.**
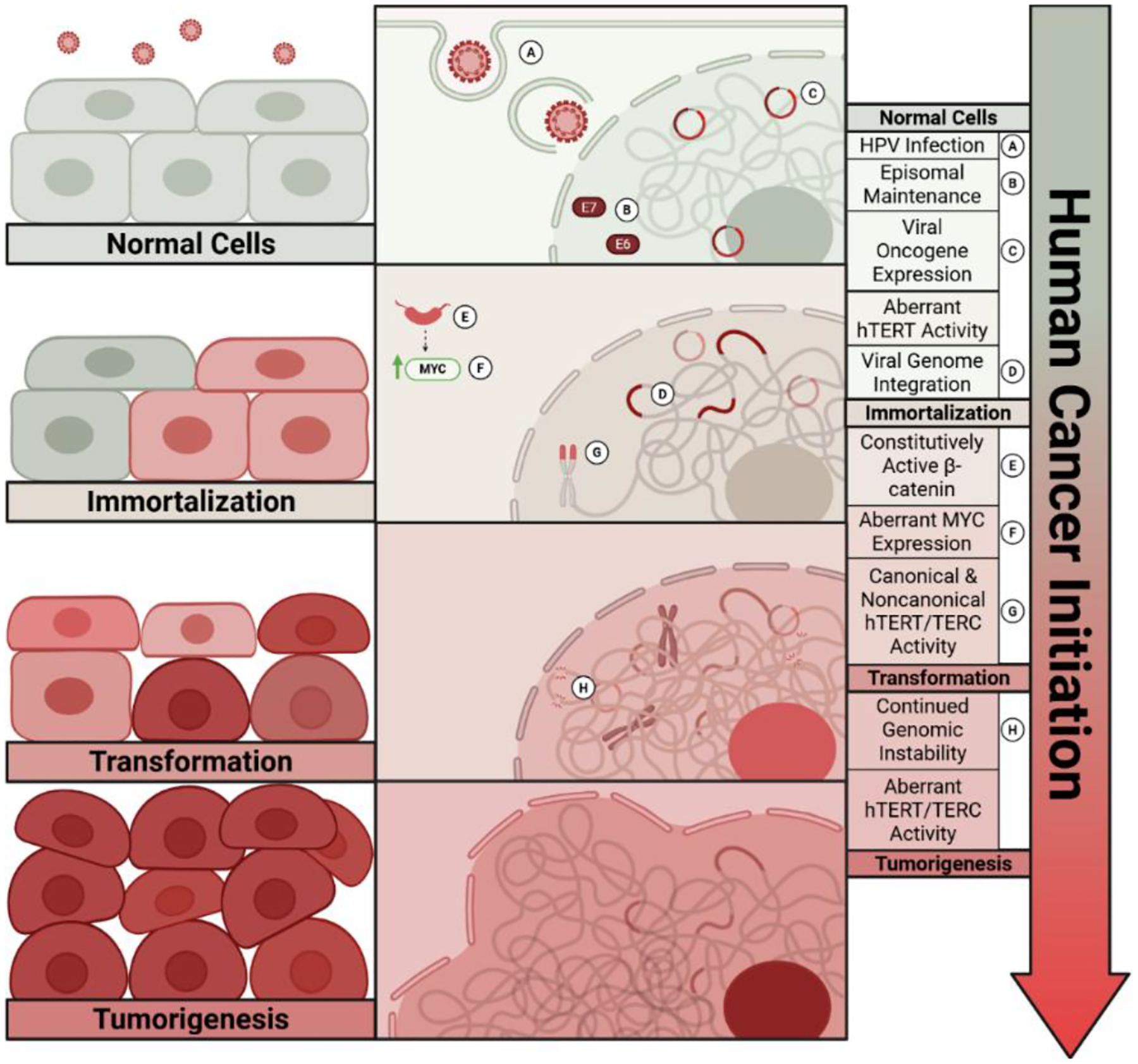
Molecular mechanisms of HPV-mediated cancer initiation. Conceptual model illustrating the proposed molecular events driving HPV-induced carcinogenesis, from initial infection through immortalization and tumorigenesis. **(A) HPV Infection:** High-risk HPV gains access to basal keratinocytes via epithelial disruptions such as micro-abrasions through persistent infections of the cervix, vagina, penis, anus, mouth, or throat. **(B) Episomal Maintenance:** The viral genome is maintained as a circular episome, replicating independently of the host genome and segregating into daughter cells during cell division. **(C) Viral Oncogene Expression:** The HPV genome contains an early promoter in the long control region (LCR) that recruits host transcriptional machinery to express viral genes, including the E6 and E7 oncoproteins. E6 targets p53 for degradation, inhibiting apoptosis, while E7 inactivates pRb, driving uncontrolled cell cycle progression. **(D) Viral Genome Integration:** While HPV oncogenes can be expressed in episomal form, integration into the host genome can disrupt both viral and host regulatory mechanisms as well as genomic integrity. This leads to increased expression of viral oncogenes, disruption of host tumor suppressor genes, and activation of host protooncogenes, promoting cellular immortalization. **(E) Constitutively Active β-catenin:** The E6 and E7 viral oncogenes stabilize β-catenin and promote nuclear translocation. From there, β-catenin functions as a transcriptional coactivator, upregulating the downstream target genes of the Wnt signaling pathway. **(F) Aberrant MYC Expression:** β-catenin-driven transcription activates the MYC proto-oncogene, which is further stabilized by E6. MYC overexpression causes uncontrolled cell cycle progression and hyperproliferation. **(G) Canonical & Noncanonical hTERT/TERC Activity:** Through a combination of previously listed mechanisms (including viral oncoprotein expression, disruption of host genome and regulatory mechanisms, and host oncogene activation), hTERT and TERC-core components of telomerase-are reactivated. Canonically, telomerase maintains telomere length, conferring replicative immortality. Noncanonically, hTERT/TERC influence gene expression, metabolism, signaling, and immune responses. These canonical and noncanonical functions work together to promote transformation [58]. **(H) Continued Genomic Instability:** These oncogenic events do not occur linearly but intersect and reinforce one another. Loss of tumor suppressors, activation of oncogenes, and sustained proliferative signaling promote genomic instability, chromosomal aberrations, and accumulation of driver mutations that culminate in tumorigenesis [43].

Figure 1 illustrates the multi-step human cell model reflecting tumor initiation with genetic alterations in cancer patients.

Together, these mechanisms highlight the multi-step processes to initiate and drive cancer progression through persistent infection, disruption of tumor suppressor pathways, genomic instability, oxidative stress, telomere dysregulation, and immune evasion, underscoring the complexity of HPV-induced carcinogenesis.

## 5. HPV Genome Integrations

Human papillomavirus (HPV) integration into the host genome represents a common but not obligatory step in cancer progression, occurring in the majority but not all HPV-associated malignancies. While a significant proportion of HPV-positive tumors maintain the virus in a strictly episomal state throughout carcinogenesis, integration events contribute substantially to tumor progression and heterogeneity when they occur and represent a pivotal transition from the episomal viral state maintained during productive infection to a genomically incorporated form that fundamentally alters both viral gene expression and host cell biology [23].

The integration process is facilitated by DNA damage response mechanisms and typically occurs at sites of double-strand breaks in both viral and host genomes [23,59]. Cellular DNA repair machinery, particularly non-homologous end joining (NHEJ) and microhomology-mediated end joining (MMEJ), inadvertently joins these broken DNA ends, resulting in stable insertion of viral sequences into host chromosomes [60]. DNA damage response proteins, including ATM, ATR, and members of the MRN complex (MRE11, RAD50, and NBS1), localize to viral replication centers and play crucial roles in facilitating integration [59,61]. Advanced sequencing technologies have revealed remarkable structural diversity within individual integration events, showing that viral DNA can exist in multiple distinct configurations at identical human genomic breakpoints. These variant integrant structures, termed heterologous configurations, occur at approximately one-fifth of integration sites and arise from differential amplification during concatemer formation [62]. The most complex integration patterns involve numerous viral-host junction points-sometimes exceeding 30 breakpoints per event-that create intricate networks of genomic rearrangements, with single breakpoints serving as central hubs that connect multiple chromosomal locations [62]. This structural complexity supports models of integration-associated genomic instability and provides mechanistic insight into the extensive chromosomal alteration characteristic of HPV-driven carcinogenesis [62]. Replication stress has increasingly been recognized as a major contributor to integration, as collisions between the replication and transcription machinery generate DNA breaks that serve as substrates for viral incorporation [63].

Two primary models have been proposed to explain the mechanistic basis of HPV integration [64–66]. The “looping” model involves DNA replication and recombination events that lead to the formation of concatemers, thereby disrupting genes associated with tumorigenesis, oncogene amplification, and genomic instability [65,67]. Complementing this is the microhomology-mediated integration model, which implicates replication-associated DNA repair mechanisms such as FoSTeS (fork stalling and template switching) and MMBIR (microhomology-mediated break-induced replication) [66,68,69]. During replication fork stalling or DNA breaks, HPV can “hijack” host repair pathways by using microhomology-rich regions between viral and host DNA to facilitate integration [69].

Integration events display remarkable heterogeneity across HPV-associated cancers. Genomic analyses have revealed that although certain fragile sites and transcriptionally active regions are preferentially targeted, the specific integration sites vary widely between tumors and even among cells within the same tumor [67]. Common fragile sites such as FRA3B (containing the FHIT gene) and FRA17B (involving the FANCD2 gene) are frequently affected, suggesting preferential targeting of genomically unstable regions [70,71]. Large-scale genomic studies have identified specific chromosomal loci that are statistically significant hotspots for viral integration. Comprehensive analysis of over 100 HPV-positive oropharyngeal tumors has identified five genomic regions with recurrent integration patterns that exceed random expectations, specifically targeting loci containing the SOX2, TP63, FGFR3, MYC, and CD274 genes [72].

**Figure 2.**
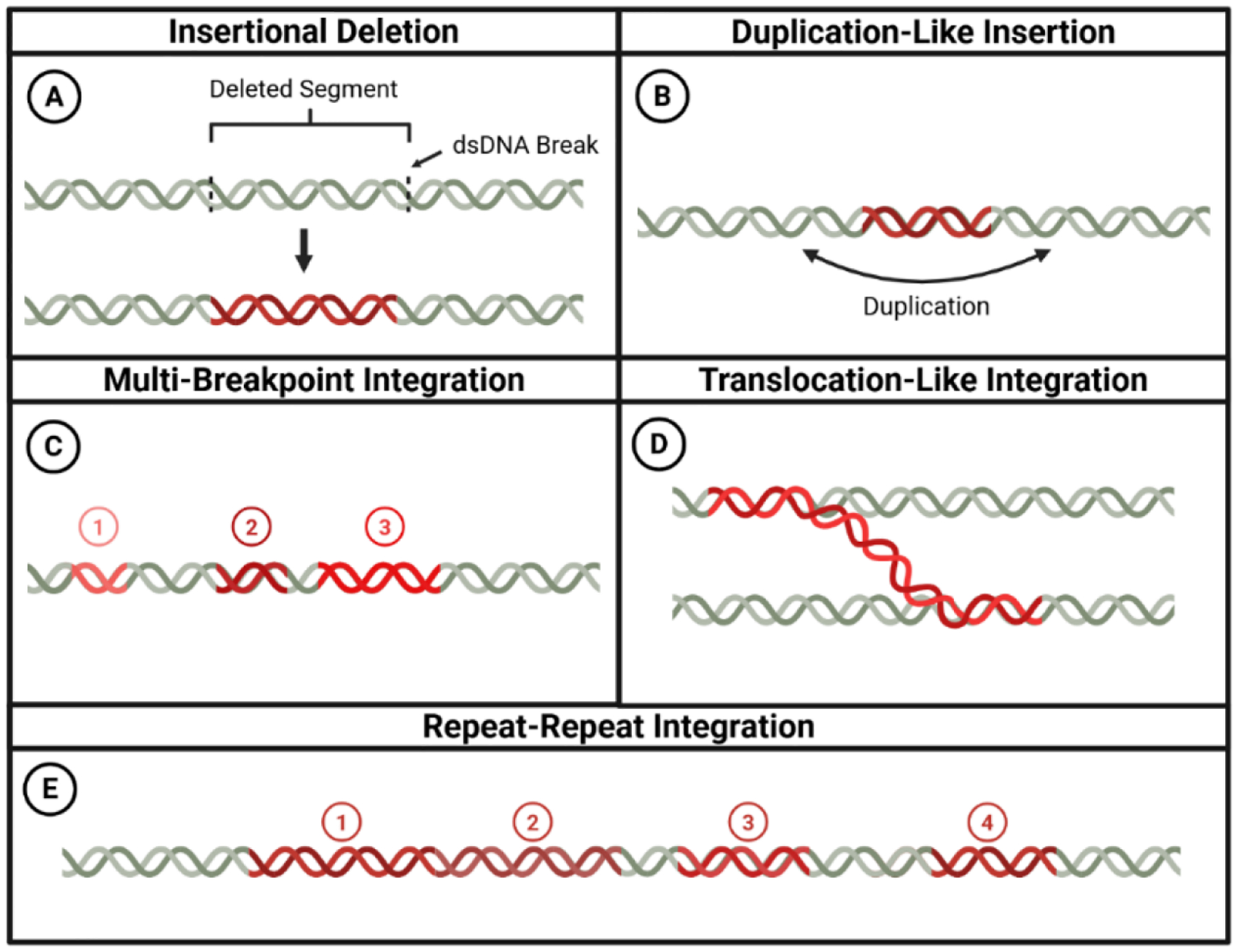
Structural variation of integration patterns in the human genome. Schematic representation of major HPV genome integration patterns as identified by long-read sequencing. **(A) Insertional Deletion:** HPV DNA (red) is inserted at two dsDNA breakpoints in the host genome (green), resulting in a deletion of the intervening host sequence. **(B) Duplication-Like Insertion:** An insertion of HPV DNA is accompanied by a duplication of adjacent host genomic sequence at the integration site. **(C) Multi-Breakpoint Integration:** An HPV integration event involving ≥3 distinct viral-host junctions within the same genomic region. **(D) Translocation-Like integration:** HPV integrates at breakpoints across two distinct chromosomal regions, acting as a bridge for chromosomal rearrangements or hybrid chromosomes. **(E) Repeat-Repeat integration:** The HPV genome integrates in highly repetitive regions of the genome, which can be viral repeats (1 & 2), host repeats, or viral-host hybrid concatemers (3 & 4).

These integration hotspots demonstrate clear functional consequences: viral insertion near MYC and CD274 correlates with gene amplification and elevated transcript levels, whereas SOX2-proximal integration events result in substantial copy number increases, accompanied by aberrant overexpression that enhances cellular proliferation capacity [72]. This genomic variability in integration patterns contributes significantly to the heterogeneous nature of HPV-driven cancers and influences their evolutionary dynamics (Figure 2).

The molecular consequences of integration are profound and multifaceted. One of the most well-characterized effects is the disruption of the viral E2 gene, which normally represses expression of the viral oncogenes E6 and E7 [60,73]. With the E2 function compromised, uncontrolled expression of these oncoproteins drives cell-cycle deregulation by interfering with the p53 and pRb tumor suppressor pathways [65,66,68,74,75]. Beyond E2 disruption, recent studies using next-generation sequencing have revealed that breakage can occur at other sites in the viral genome, including the E1 gene, indicating multiple forms of viral genome alteration can contribute to deregulated oncogene expression [65,68].

Integration events can also generate oncogenic host gene fusions through the same heterocateny-driven processes that create other forms of structural variation [76]. Long-read sequencing analyses reveal that unstable virus concatemers facilitate the capture and rearrangement of flanking host DNA sequences, leading to the formation of in-frame gene fusions such as FGFR3-TACC3 [77]. Functional studies demonstrate that these integration-derived fusions can cooperate with viral oncoproteins to drive malignant transformation, with co-expression of HPV16 E6/E7 and FGFR3-TACC3 fusions sufficient for tumor formation in mouse models, whereas neither component alone can induce tumors [77]. This represents the first direct evidence that the complex genomic rearrangements characteristic of heterocateny can generate functionally essential oncogenic drivers. Integration affects more than viral gene expression; it also affects host genomic stability. The insertion of viral sequences can directly impact host genes at or near integration sites through insertional mutagenesis, oncogene amplification, or formation of viral-host fusion transcripts with novel functions [78,79]. Two principal mechanisms have been identified through which HPV integration leads to carcinogenesis: 1) tumor suppressor gene inactivation, and 2) oncogene upregulation through promoter insertion, gene amplification, or enhancer effects [68,80]. In the first mechanism, integration into tumor suppressor genes such as RAD51B compromises DNA repair fidelity [78,81]. The second mechanism involves viral integration near or upstream of oncogenes like NR4A2, FOXE1, PIM1, or the MYC locus, leading to their overexpression. Additionally, integration can induce complex inter- and intra-chromosomal rearrangements affecting genes like TRPG1, TP63, and KLF5 [68,78]; however, these rearrangements ultimately exert their oncogenic effects by disrupting tumor suppressor function or activating oncogenic pathways, rather than representing a distinct mechanistic category.

Integration-mediated formation of viral-host fusion transcripts represents a particularly interesting phenomenon with significant biological implications. A diverse array of viral-host fusion transcripts has been characterized in HPV-positive head and neck cancers, often exhibiting enhanced stability due to alterations in 3’ untranslated regions [82]. These chimeric RNAs may encode novel proteins with altered functions or disrupt normal gene expression patterns [67,82]. Recurrent HPV integration at loci such as RAD51B in cervical cancers further highlights the potential for integration to exacerbate genomic instability through disruption of DNA repair genes [68].

Beyond localized effects at integration sites, HPV integration triggers broader genomic instability through multiple mechanisms. The integration process itself can induce complex structural variations in host chromosomes, including deletions, amplifications, and translocations [65,67,83]. Furthermore, persistent expression of viral oncoproteins such as E6 and E7 exacerbates the mutational burden by interfering with DNA damage repair pathways [68,78,80]. E6-mediated degradation of p53 compromises cell cycle checkpoints, while E7-mediated disruption of the retinoblastoma family proteins promotes aberrant cell cycle progression [84]. This genomic instability creates a permissive environment for rapid tumor evolution and the emergence of diverse subclones with varied biological properties.

The APOBEC family of cytidine deaminases has emerged as an important contributor to mutagenesis following HPV integration. Enzymes such as APOBEC3A and APOBEC3B induce cytidine deamination events in both host and viral DNA, generating mutation-prone sites that may facilitate additional integration events or fuel tumor evolution [85]. Distinctive APOBEC mutation signatures have been consistently observed in HPV-positive malignancies, underscoring their ongoing role in shaping the mutational landscape post-integration [85,86].

Recent studies have also demonstrated important connections between HPV integration and epigenetic reprogramming. Methylation patterns tend to increase in the later stages of cervical carcinogenesis and correlate with viral genomic state [87,88]. Aberrant methylation particularly affects the upstream regulatory region (URR), including E2-binding sites, as well as the L1 and L2 regions [65,85,87]. These epigenetic changes correlate with disease progression and can lead to deregulation of E6/E7 expression [65,89].

Advances in single-cell sequencing technologies have revealed that HPV integration is not always a clonal event, particularly in early lesions. Sub-clonal integration contributes to intra-tumoral heterogeneity and provides the genetic diversity necessary for tumor evolution [90]. Analysis of integration patterns has shown that single integration events shared across all tumor cells suggest early integration followed by clonal expansion, whereas heterogeneous integration patterns indicate ongoing integration or the expansion of multiple distinct clones [65,91]. These extrachromosomal structures exhibit characteristics typical of oncogenic ecDNA, including high copy number, unequal partitioning to daughter cells, and open chromatin architecture that promotes high levels of gene expression [92]. This clonal architecture, shaped by integration events, influences tumor progression and potentially therapeutic response.

Technological advancements have greatly improved the ability to detect and characterize HPV integration events. Traditional PCR-based methods and fluorescence in situ hybridization (FISH) have been supplemented by next-generation sequencing techniques, such as whole-genome and RNA sequencing, to detect viral-host fusion transcripts. More recently, long-read sequencing technologies, including Oxford Nanopore and PacBio, have enabled the comprehensive mapping of complex integration structures [83,93]. At the single-cell level, DNA and RNA sequencing platforms now provide unprecedented resolution, revealing the interplay between viral integration events, transcriptional programs, and clonal architecture [69].

Emerging evidence suggests that HPV integration status correlates with prognosis and treatment response in HPV-associated cancers. Integration appears more frequent in advanced disease stages [81]; however, the presence of integrated HPV has also been detected in early cervical intraepithelial neoplasia (CIN) lesions [94,95]. Patients with a mixed viral state (both episomal and integrated forms) appear to have a higher probability of progression compared to those with exclusively integrated or exclusively episomal viral genomes [95]. The dynamic nature of these integration-mediated rearrangements suggests that heterocateny may drive tumor evolution and heterogeneity, though the prognostic significance of these complex genomic alterations in HPV-positive cancers remains to be determined [92]. Thus, viral states may serve as biomarkers of disease trajectory.

**Figure 3.**
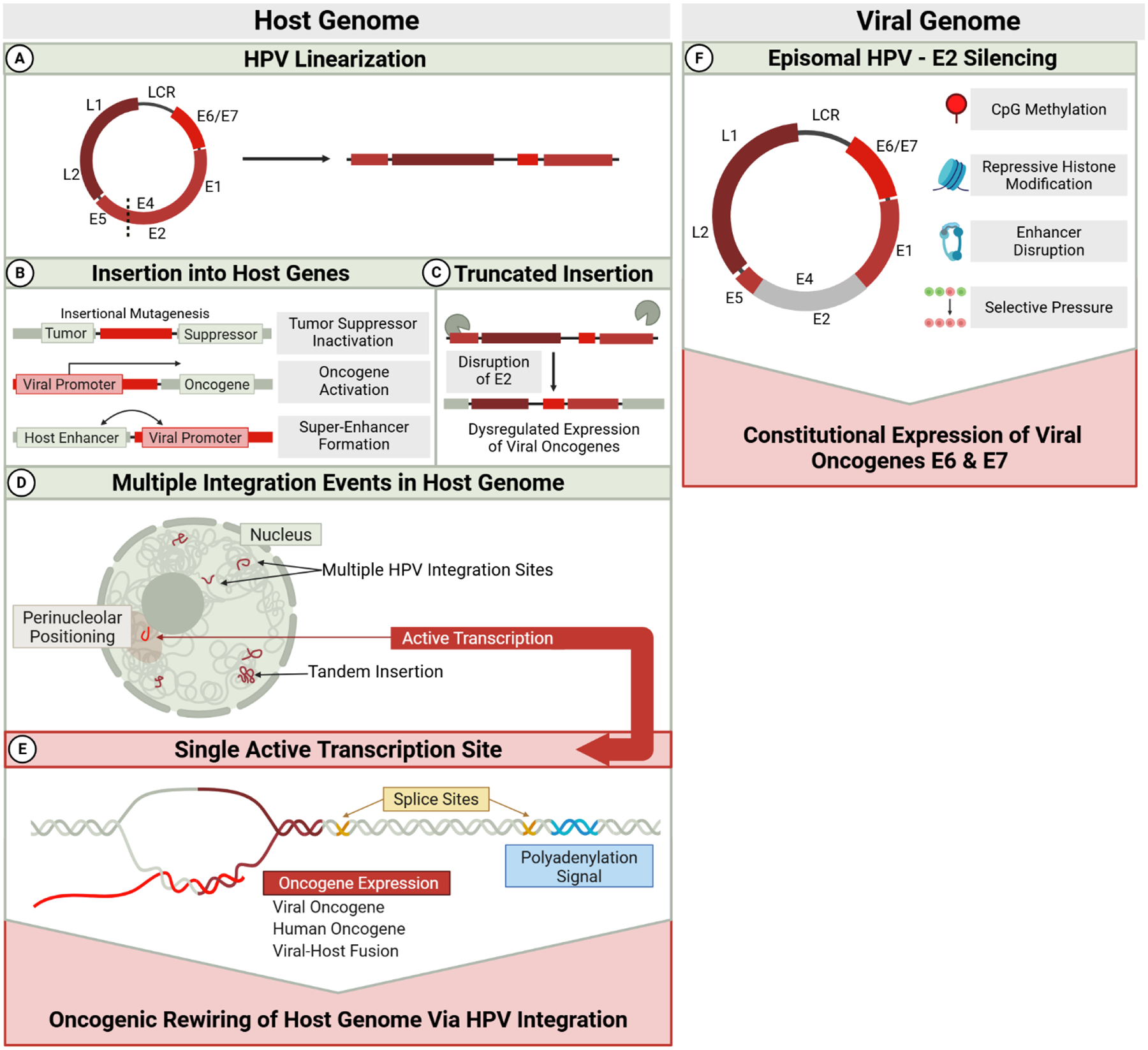
Dysregulated E6/E7 expression from integrated and episomal HPV genomes. Schematic representation of how high-risk human papillomavirus (HPV) drives aberrant expression of the viral oncogenes E6 and E7 via episomal persistence (right) and integration into the host genome (left). **(A) HPV Linearization:** The circular HPV genome is linearized, typically by disrupting the E1/E2 region, enabling integration into the host genome. **(B) Insertion into Host Genes**: Integration can occur within **tumor suppressor** genes, leading to their inactivation, or near **oncogenes**, enabling their cis-activation by viral promoters. Integration at or near host **super-enhancers** can also amplify viral transcription. **(C) Truncated Insertion:** Prior to insertion, the linearized HPV genome is subject to host 3’ and 5’ nucleases. Although how truncation occurs is not yet established, these nucleases are hypothesized to degrade the viral genome before integration. If the E2 suppressor region of the HPV genome is lost to degradation, E6 and E7 oncogenes will be overexpressed. **(D) Multiple Integration Events in the Host Genome:** HPV integrates at multiple genomic loci, often in complex, rearranged concatemeric forms involving viral and host DNA. Despite multiple insertions, a single transcriptionally active integration site typically dominates, often localized to the perinucleolar region. **(E) Single Active Transcription Site:** Active viral transcription occurs at a locus where HPV sequences (lacking E2) are adjacent to functional splice sites and a host polyadenylation signal (PAS), enabling stable oncogene expression through viral, host, or fusion transcripts. **(F) Episomal HPV - E2 Silencing:** The unregulated expression of E6 and E7 oncoproteins can occur without integration by the silencing of episomal E2. This is accomplished via CpG methylation, histone modifications, enhancer disruption, or selection for escape variants. Each pathway converges on transcriptional activation of viral oncogenes, driving malignant transformation. Red boxes summarize key oncogenic consequences of each mechanism.

This integration-driven heterogeneity provides a foundation for subsequent exploration of cellular and molecular heterogeneity using single-cell approaches. The complex interplay between viral integration, genomic instability, and clonal evolution represents a critical aspect of HPV oncogenesis that warrants continued investigation to enhance our understanding of tumor biology and improve the management of HPV-associated malignancies. Interestingly, despite heterogeneous HPV integration patterns in the host genome described above, only one active transcription site at the HPV end of all integrated viral genomes contributes to sustained expression of the E6 and E7 oncogenes [96,97](Figure 3), a hallmark of nearly all HPV-positive cancers.

## 6. Single-Cell RNA Sequencing: Cell/HPV Protein Based Molecular Heterogeneity

The complex heterogeneity of HPV-induced cancers necessitates advanced detection strategies. Traditional bulk RNA sequencing captures only average gene expression profiles and is therefore limited in its ability to resolve cellular diversity relevant to HPV-driven tumor biology. In contrast, single-cell RNA sequencing (scRNA-seq) provides a comprehensive, high-resolution view of tumor ecosystems, enabling spatial and temporal analyses, and offering new opportunities to improve diagnostics and therapeutic approaches. Here, we outline how scRNA-seq helps address challenges in diagnosis, prognosis, treatment response, prevention, and disease monitoring in HPV-associated cancers by facilitating the characterization of tumor microenvironment features, oncogene expression patterns, precancerous lesions, and their progression to malignancy.

A key application of scRNA-seq is its ability to characterize the tumor microenvironment (TME), a major determinant of therapeutic response. Single-cell sequencing overcomes the limitations of traditional bulk sequencing by enabling the identification and resolution of small cellular subsets within tumor tissues. This approach provides insight into the composition of the TME and HPV-associated cellular evolution, both of which are critical to understanding the development of treatment resistance. For example, in one scRNA-seq study analyzing cervical cancer (CC) tissues, live cells from tumors and adjacent normal samples were classified into epithelial, endothelial, and macrophage populations. Notably, ABC transporters were highly expressed in the endothelial subpopulation, with significantly higher expression in tumorderived endothelial cells compared with normal counterparts. Given the established roles of ABC transporters in angiogenesis and chemoresistance, these findings suggest that endothelial cells may contribute to CC angiogenesis and therapy resistance, and that ABC transporters could represent potential prognostic markers and therapeutic targets [98].

Immune cells constitute another critical component of the TME. Immune dysfunction is a major driver of tumor immune escape during initiation, and tumor progression further suppresses host immunity, resulting in cancer-type-specific microenvironments that provide valuable opportunities for therapeutic intervention [99]. A study integrating scRNA-seq with T-cell receptor sequencing in CD4^+^ and CD8^+^ cells revealed upregulation of CD4^+^ regulatory T cells in the CC TME, consistent with an immunosuppressive milieu. CD8^+^ T cells, which are essential for recognizing and eliminating malignant cells, were reduced, and those remaining frequently exhibited an exhausted phenotype characterized by elevated checkpoint gene expression, indicating tumor-mediated immune evasion through checkpoint pathways. These findings highlight the potential utility of immune checkpoint blockade therapies in cervical cancer [100,101].

Moreover, scRNA-seq enables molecular comparisons between HPV-positive and HPV-negative squamous cell carcinomas (SCCs), facilitating investigation of prognostic heterogeneity. One study reported that HPV-negative samples recruited fewer epithelial cells but more T cells, suggesting distinct immune dynamics between HPV-positive and HPV-negative tumors [102]. Consistently, recent transcriptomic profiling demonstrates that HPV-positive CSCC cells adopt more differentiated cancer states, whereas HPV-negative CSCC cells display reduced differentiation and increased stemness. In addition, HPV-negative tumors exhibit higher immune infiltration, underscoring biologically distinct tumor ecosystems with important implications for prognosis and treatment strategies [103].

scRNA-seq also provides a powerful framework for addressing viral gene-specific questions. To investigate interactions between HPV oncogenes and epithelial differentiation states, expression patterns of HPV16 genes (E1, E6, and E7) were examined across epithelial clusters. Nine clusters were identified as HPV-positive to varying degrees, and clusters with the highest HPV gene expression exhibited distinct functional and structural characteristics compared with normal epithelial cells, indicating that viral oncogenes can remodel epithelial architecture [5].

Beyond viral transcriptional profiling, scRNA-seq enables detailed characterization of both intratumoral and intertumoral heterogeneity. In a study of 16 OPSCC tumors, samples were initially classified as HPV-positive or HPV-negative based on epithelial genotypes. Subsequent classification of tumors as malignant or non-malignant based on chromosomal copy-number aberrations (CNAs) revealed substantial heterogeneity within individual tumors and suggested malignant potential even in HPV-negative cells, highlighting the limitations of categorizing tumors solely by HPV gene expression [104].

Focusing on HPV integration patterns, scRNA-seq further revealed marked intratumoral heterogeneity. Based on cancer cell fraction, 44% of integration breakpoints were identified as subclonal. In contrast to clonal populations, in which breakpoints predominantly occurred within the E1 region, subclonal breakpoints were enriched in the L1 and E6 regions. By integrating breakpoint data with HPV genome copy number, four HPV physical states were defined, and the results indicated that viral integration is not strictly required for carcinogenesis. Additional analyses of cancer-associated genes showed that nonsynonymous-to-synonymous substitution ratios identified PI3K mutations as a major driver of tumorigenesis, while JAK-STAT and NF-κB signaling pathways contributed to specific subpopulations [105].

Beyond microenvironmental and viral profiling, scRNA-seq enables investigation of tumor progression by resolving cell-intrinsic programs independent of bulk tissue effects, providing insight into how precancerous lesions evolve into malignant disease [98]. In cervical cancer tissue, comparative transcriptomic analysis between high-grade squamous intraepithelial lesions (HSIL) and other cell types revealed enrichment of C2-Ma-THBS1 macrophages in HSIL, whereas C1-Ma-C1QA macrophages predominated in tumor cells. Pathway analysis implicated IL-1, NF-κB, and cytokine-mediated signaling in this transition, supporting their roles in tumor progression [100].

Complementary scRNA-seq studies examining spatial and temporal dynamics of lesion progression identified three upregulated genes (SPRR3, CEACAM7, APOBEC3A) and three downregulated genes (TCN1, TFF3, BPIFB1) in HSIL, suggesting potential roles as lesion-promoting or protective factors and highlighting novel therapeutic opportunities to prevent malignant transformation [106]. In HPV-negative head and neck squamous cell carcinoma (HNSCC), analysis of DNA copy-number alterations in precancerous leukoplakia revealed carcinoma in situ cells that were undetectable by conventional pathology. TP63 and ATP1B3, which showed the most significant CNA-associated downregulation in these cells, were subsequently validated as markers of HNSCC progression [107]. Collectively, these findings demonstrate the power of scRNA-seq to delineate HPV-associated tumor microenvironments, oncogene-driven transcriptional programs, and disease progression, providing actionable insights for early diagnosis and therapeutic intervention.

## 7. The “Hit and Run” Hypothesis in HPV-Associated Cancers

The “hit and run” hypothesis proposes that human papillomavirus (HPV) may initiate carcinogenesis through its oncoproteins E6 and E7, which disrupt p53 and Rb tumor suppressor pathways, but may later become dispensable as the tumor accumulates independent genomic alterations [108]. Unlike cervical cancers, where persistent HPV oncogene expression maintains malignancy, emerging evidence shows that 5–15% of HPV-driven oropharyngeal carcinomas progress to a virus-independent state through either complete HPV DNA loss or epigenetic silencing of viral genes [109,110]. This ‘hit and run’ phenomenon is particularly observed in metastatic and recurrent tumors, where the viral genome becomes undetectable despite the cancer maintaining its aggressive phenotype [108]. This phenomenon suggests that early viral activity (“hit”) induces irreversible genomic instability, including telomerase activation [111], aneuploidy from centrosome duplication errors [112], and epigenetic dysregulation [113], which ultimately allows the tumor to progress without ongoing viral dependence (“run”). Supporting this, whole-exome sequencing reveals that HPV^−^ head and neck squamous cell carcinomas (HNSCCs) harbor TP53 mutations and CDKN2A deletions far more frequently than HPV^+^ tumors, implying that host mutations can substitute for viral oncogenic drivers [114]. Clinically, 15–20% of HPV-associated oropharyngeal cancers lose detectable HPV DNA in metastatic lesions [115]. However, debates persist over how often true “hit and run” occurs, with estimates ranging from 5% to 30%, depending on detection methods, and whether viral loss occurs early during premalignant progression or later during metastasis [116]. This has direct diagnostic and therapeutic implications: p16 immunohistochemistry, a common HPV surrogate, can misclassify virally initiated but HPV-independent tumors [117], and such tumors may resist therapies targeting viral antigens, like therapeutic vaccines [118]. Emerging solutions include multimodal biomarker strategies that combine viral DNA detection, integration site analysis, and host mutation profiling, as well as therapies targeting both viral and acquired host vulnerabilities [34]. Current research leverages single-cell multiomics to trace clonal evolution following viral loss and to refine treatment paradigms for these biologically distinct cancers.

## 8. Combined Therapies to Overcome Heterogeneity and Resistance

**Figure 4.**
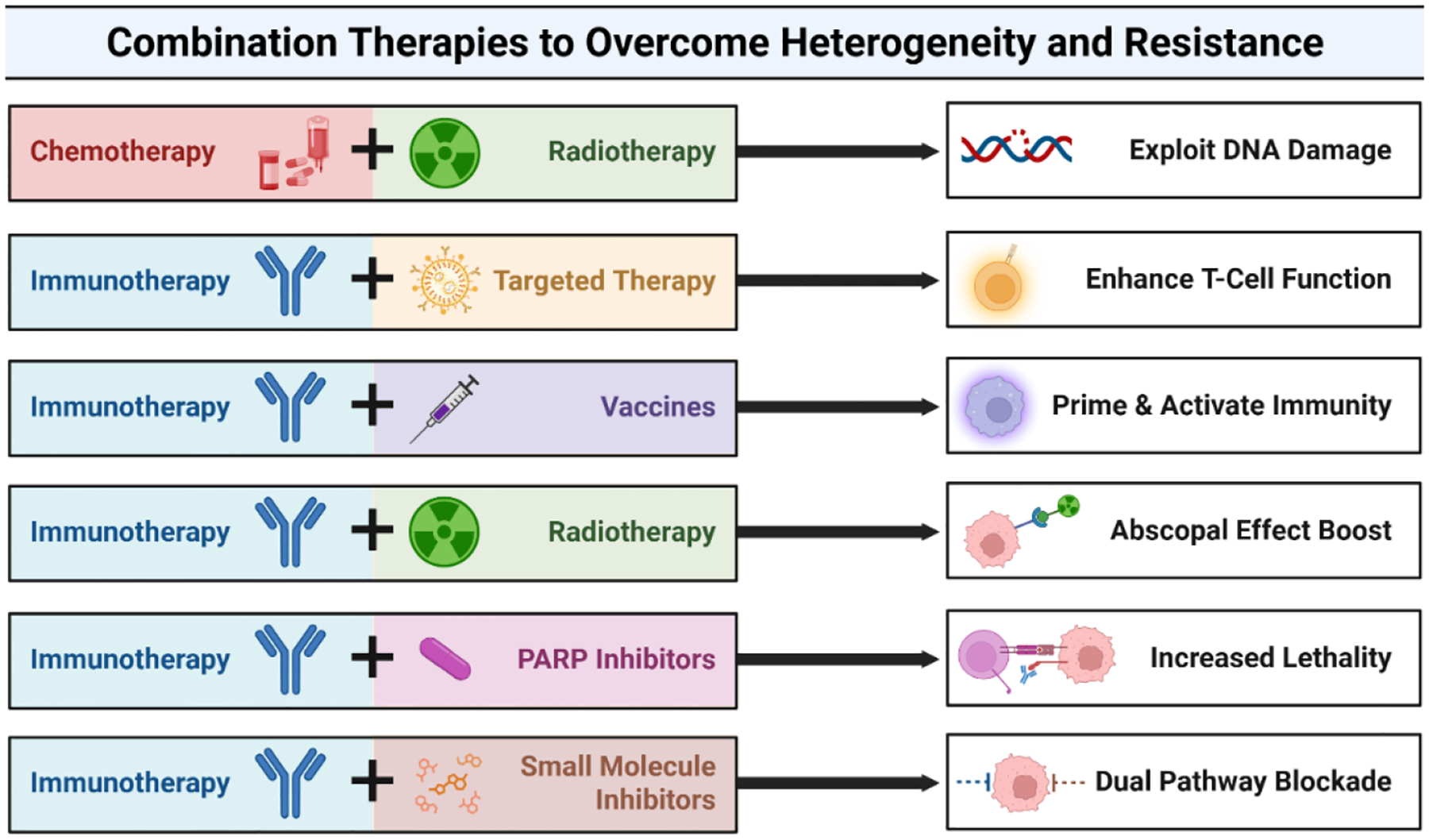
Combination therapy strategies to overcome tumor heterogeneity and resistance. The synergistic cancer treatment combinations are designed to address tumor heterogeneity and therapy resistance. Each row pairs a therapeutic strategy with its biological goal or benefit, highlighting mechanisms such as immune enhancement, pathway blockade, and induction of systemic antitumor effects.

Human papillomavirus (HPV)-associated cancers-including cervical, oropharyngeal, and anal malignancies-are primarily driven by the viral oncoproteins E6 and E7, which inactivate key tumor suppressors such as p53 and Rb [119]. These disruptions foster genomic instability, facilitate immune evasion, and dysregulate key cellular signaling pathways. However, the therapeutic response varies greatly due to molecular heterogeneity in viral integration, host genetics, and immune landscape [120]. Consequently, combined therapies targeting multiple vulnerabilities have emerged as a strategic approach to improve clinical outcomes by overcoming the heterogeneity of HPV-related tumors (Figure 4 and Table 1).

### 8.1. Chemotherapy + Radiotherapy

HPV^+^ tumors are particularly sensitive to DNA-damaging treatments because E6/E7 impair DNA repair mechanisms [121,122]. This has been exploited in regimens such as concurrent cisplatin-based chemoradiotherapy, improving local control and survival in advanced cervical and oropharyngeal cancers [123]. Clinical trials such as NRG Oncology RTOG 1016 have confirmed the effectiveness of this approach, showing comparable efficacy between cisplatin- and cetuximab-based chemoradiation, though cisplatin remains the standard in HPV-positive cases [124]. Similarly, the TPF (docetaxel-cisplatin-5FU) induction protocol followed by radiation has yielded high 3-year survival rates (>85%) in HPV-positive head and neck cancer patients [124].

### 8.2. Immunotherapy + Targeted Therapy

Immune checkpoint inhibitors (ICIs) have revolutionized the treatment of HPV-related malignancies due to the viral antigen-driven immunogenicity of these tumors [125]. However, immune evasion and low tumor immunogenicity in some patients necessitate combinatorial strategies. Targeted therapies such as EGFR inhibitors can improve T-cell infiltration and antigen presentation [126], thereby enhancing the efficacy of ICIs. The KEYNOTE-048 trial demonstrated pembrolizumab’s superiority over chemotherapy in PD-L1-positive head and neck squamous cell carcinoma (HNSCC), showing significant improvements in overall survival (median 14.9 months *vs*. 10.7 months; HR 0.61; p<0.001) compared to cetuximab combined with chemotherapy [127], accompanied by the caveat that unlike previous smaller trials, phase III data (*e.g*. KEYNOTE-048) has not shown a clear preferential response to immunotherapy in the HPV-positive as opposed to the HPV-negative head & neck tumor subgroup. Meanwhile, the combination of balstilimab (anti-PD-1) and zalifrelimab (anti-CTLA-4) showed promising efficacy in recurrent and metastatic cervical cancer in this phase II trial [128]. However, future studies should stratify patients by HPV integration status, DDR mutations, and immune gene signatures to tailor combination regimens.

### 8.3. Therapeutic Vaccines + Immunotherapy

Therapeutic vaccines that target HPV E6/E7 aim to stimulate tumor-specific immune responses. However, their efficacy alone is limited by the suppressive tumor microenvironment. Combining vaccines with ICIs overcomes these limitations by priming and activating T cells. In the phase II trial by Massarelli et al., the combination of a DNA vaccine with PD-1 blockade achieved a 45% objective response rate in incurable HPV-16 cancers [125]. The VGX-3100 therapeutic vaccine demonstrated efficacy in cervical intraepithelial neoplasia, with 49.5% of patients receiving VGX-3100 achieving histologic regression, compared with 30.6% in the placebo group in the phase 2b trial [126].

### 8.4. Radiotherapy + Immunotherapy

Radiotherapy not only kills tumor cells but also modulates the tumor microenvironment by releasing tumor antigens and promoting immunogenic cell death [130]. Although the abscopal effect, in which localized radiation induces systemic antitumor immunity, has been observed, it remains relatively rare due to interpatient variability in immune responses [131]. Combining radiotherapy with ICIs enhances this effect by amplifying immune activation and overcoming microenvironmental suppression. This approach is particularly relevant in HPV-related cancers, where radiotherapy-induced antigen release can synergize with immune checkpoint blockade [132]. In a clinical trial by Mehra et al., the safety and efficacy of pembrolizumab were evaluated in patients with advanced HNSCC who had prior platinum-based chemotherapy. The study focused on long-term outcomes, including overall response rate (ORR) and progression-free survival (PFS). The ORR was 18%, with a median PFS of 2.1 months and a median overall survival of 8 months (38% OS at 12 months). PD-L1 expression has been shown to correlate with treatment efficacy, particularly in HPV-associated HNSCC patients [133]. The E1308 Phase II trial results showed that patients with a clinical complete response (cCR) to chemotherapy had an 80% progression-free survival (PFS) rate and a 94% overall survival (OS) rate with reduced radiation, along with significantly fewer swallowing and nutritional issues. The study suggests that radiation de-escalation is a viable approach for certain patients, particularly those with low smoking history and HPV-positive OPSCC, to reduce treatment-related toxicities while maintaining effective disease control [7]. Trials such as PEMBRO-RT showed improved outcomes when pembrolizumab was combined with stereotactic body radiotherapy (SBRT) in patients with metastatic non-small cell lung cancer (NSCLC) [10], and similar approaches are being applied to HPV-related cancers.

### 8.5. PARP Inhibitors + Immunotherapy

HPV-induced defects in p53 and other DNA repair genes may sensitize tumors to poly (ADP-ribose) polymerase (PARP) inhibitors [119]. However, resistance may develop through compensatory pathways or immune escape. Combining PARP inhibitors with ICIs can increase neoantigen load and enhance immune recognition [8]. A study by Kono et al. demonstrated significantly increased activation of DNA damage repair factors such as FANCD2, BRCA1, and γH2AX in HPV^+^ oropharyngeal cancers compared to HPV^−^ ones, suggesting that HPV^+^ tumors, with their underlying DNA repair deficiencies, may be particularly susceptible to therapies such as PARP inhibition [9]. The MEDIOLA trial reported synergistic activity of olaparib and durvalumab in BRCA-mutant ovarian cancer, highlighting the potential of this combination strategy in tumors with genomic instability, such as those found in certain HPV-related cancers [134].

### 8.6. Small Molecule Inhibitors + Immunotherapy

Oncogenic signaling pathways, such as PI3K/AKT/mTOR, are frequently dysregulated in HPV-related cancers due to host genomic alterations and viral oncoprotein activity [6]. Small-molecule inhibitors targeting these pathways can suppress tumor growth but are often insufficient to eliminate genetically heterogeneous tumor cell populations [11]. Combining these inhibitors with ICIs addresses both oncogenic signaling and immune evasion, creating a more comprehensive therapeutic strategy [12].

The unique molecular features of HPV-associated cancers, including viral antigen expression and DNA repair defects, provide multiple opportunities for rational combination therapies. Current evidence supports chemoradiation as the standard backbone therapy for localized disease, while immunotherapy-based combinations are increasingly promising for recurrent or metastatic HPV-positive cancers.

## Figures and Tables

**Table 1.** Combination therapies under investigation for HPV-associated cancers, with underlying biological rationale and representative clinical trials.

Combination Type	Biological Rationale / Mechanism	Representative Clinical Trial(s)	Key Notes / Outcomes
**Chemoradiotherapy (Cisplatin + Radiation)**	DNA damage from radiation synergizes with cisplatin; HPV^+^ tumors show increased radiosensitivity due to impaired DNA repair from E6/E7 activity.	RTOG 1016, NCT01302834	Cisplatin + radiation remains SOC; cetuximab + radiation inferior for HPV^+^ OPSCC
**Immunotherapy (PD-1/PD-L1 Blockade ± Chemotherapy)**	Restores antitumor T-cell function suppressed by PD-1/PD-L1 signaling; HPV antigens enhance immunogenicity.	KEYNOTE-048 (NCT02358031), NCT02255097	Pembrolizumab ± chemo improved OS *vs* cetuximab + chemo in recurrent/metastatic HNSCC.
**PD-1 + CTLA-4 Dual Blockade**	Synergistic activation of T-cell priming and effector phases to overcome immune exhaustion.	NCT02369874	Balstilimab + Zalifrelimab: ~26% ORR in advanced CC; manageable toxicity.
**Therapeutic DNA Vaccine (VGX-3100) + PD-1 Inhibitor**	Vaccine primes HPV-specific T cells; PD-1 blockade prevents suppression within the TME.	NCT03721978	DNA vaccine + pembrolizumab showed ~45% ORR in incurable HPV-16^+^ cancers.
**Radiotherapy + Immunotherapy**	Radiation induces immunogenic cell death, antigen release, and abscopal effects enhanced by checkpoint blockade.	E1308, NCT02684253 (Pembro-RT)	De-escalated RT + ICI improved PFS/OS in HPV^+^ OPSCC; ongoing optimization.
**PARP Inhibitor (Olaparib) + PD-L1 Inhibitor (Durvalumab)**	HPV-induced DDR defects sensitize to PARP blockade; increases neoantigen load to enhance immune recognition.	MEDIOLA (NCT02734004)	Proof of concept for DDR-defective, HPV^+^ tumors; evaluating cervical and HNSCC cohorts.
**EGFR Inhibitor (Erlotinib/Cetuximab) + PD-1 Inhibitor**	EGFR inhibition augments antigen presentation and T-cell infiltration; counteracts immune-cold phenotype.	NCT03082534, NCT03370276	Under investigation; rationale for HPV^+^ HNSCC with EGFR activation.
**Cisplatin + Immune Checkpoint Inhibitor**	Cisplatin induces immunogenic cell death and MHC-I upregulation; potentiates ICI efficacy.	NCT03894215, NCT04576091	Evaluating concurrent ICI during CRT for locally advanced CC and HNSCC.

## Data Availability

Data sharing is not applicable to this article as no datasets were generated or analyzed during the current study.

## Abbreviations

The following abbreviations are used in this manuscript:

CC: Cervical Cancer
HNSCC: Head and Neck Squamous Cell Carcinoma
ICI: Immune Checkpoint Inhibitor
DDR: DNA Damage Repair
ORR: Objective Response Rate
OS: Overall Survival
PFS: Progression-Free Survival
SOC: Standard of Care
TME: Tumor Microenvironment
